# Artificial Intelligence in bone Metastases: A systematic review in guideline adherence of 92 studies

**DOI:** 10.1016/j.jbo.2025.100682

**Published:** 2025-04-24

**Authors:** Lotte R. van der Linden, Ioannis Vavliakis, Tom M. de Groot, Paul C. Jutte, Job N. Doornberg, Santiago A. Lozano-Calderon, Olivier Q. Groot

**Affiliations:** a Department of Orthopaedic Surgery, University Medical Center Groningen, Groningen, the Netherlands; b Department of Orthopaedic Surgery, Massachusetts General Hospital, Boston, MA, USA; c Department of Orthopaedic Surgery, University Medical Center Utrecht, Utrecht, the Netherlands

**Keywords:** Metastatic bone disease, Machine Learning, Artificial Intelligence, Systematic Review, Guidelines

## Abstract

**Background:**

The last decade has witnessed a surge in artificial intelligence (AI). With bone metastases becoming more prevalent, there is an increasing call for personalized treatment options, a domain where AI can greatly contribute. However, integrating AI into clinical settings has proven to be difficult. Therefore, we aimed to provide an overview of AI modalities for treating bone metastases and recommend implementation-worthy models based on TRIPOD, CLAIM, and UPM scores.

**Methods:**

This systematic review included 92 studies on AI models in bone metastases between 2008 and 2024. Using three assessment tools we provided a reliable foundation for recommending AI modalities fit for clinical use (TRIPOD or CLAIM ≥ 70 % and UPM score ≥ 10).

**Results:**

Most models focused on survival prediction (44/92;48%), followed by imaging studies (37/92;40%). Median TRIPOD completeness was 70% (IQR 64–81%), CLAIM completeness was 57% (IQR 48–67%), and UPM score was 7 (IQR 5–9). In total, 10% (9/92) AI modalities were deemed fit for clinical use.

**Conclusion:**

Transparent reporting, utilizing the aforementioned three evaluation tools, is essential for effectively integrating AI models into clinical practice, as currently, only 10% of AI models for bone metastases are deemed fit for clinical use. Such transparency ensures that both patients and clinicians can benefit from clinically useful AI models, potentially enhancing AI-driven personalized cancer treatment.

## Highlights

• Clinically integrating AI to enhance personalized treatment has been difficult.
• Nine out of 92 AI models were fit for clinical use.
• Promoting guideline adherence is vital to establishing clinically useful AI models.
• Essential to prioritize global collaboration on data storage and processing.

## 1 Introduction

In recent years, the integration of artificial intelligence (AI) into the medical sector has shown great promise [[1], [2], [3], [4]]. The challenge of metastatic bone disease is a significant burden on both patients and society, with its prevalence anticipated to increase due to the increasing incidence and prolonged survival of cancer patients [5]. Bone metastases, often linked with a poor prognosis, leads to severe complications such as pain, immobility, and other skeletal related events, highlighting a focus on palliative care [6].

Navigating end-of-life treatment decisions can be challenging. AI may offer considerable benefits by enabling personalized medicine and aiding shared decision-making, particularly in prognostication [1,7]. Prior to the advent of AI driven prognostic models, various statistical models were devised to assist physicians in providing personalized advice for their patients. In orthopaedic oncology, the focus of these prognostic models has predominantly been on survival prediction. Prediction of survival aids clinicians in decision-making and helps tailor cancer treatment to the patient’s wishes [[8], [9], [10], [11], [12]]. These statistical models, commonly relying on regression methods, provide clear insights into the relationship between input and output variables, making them easy to interpret and therefore attractive for physicians [[8], [9], [10], [11], [12], [13]]. However, they lack the self-learning capabilities inherent in modern AI approaches. AI models stand out for their ability to sift through vast databases, detecting complex patterns and relations. Yet, their complex, non-linear architecture and opacity often render them “black boxes”, making it challenging for clinicians to decipher [14].

When discussing AI, which serves as an umbrella term for various methods, it is essential to define key concepts such as Machine Learning (ML) and Deep Learning (DL). ML, a subset of AI, focuses on developing algorithms that learn from data and make predictions without needing explicit programming. DL, a more advanced type of ML, uses multi-layered neural networks to process complex data. DL is especially useful in medical imaging aiding in bone metastasis diagnosis, object detection, and lesion segmentation. DL techniques, convolutional neural networks (CNNs) are especially effective for analyzing medical images. By training on specialized or diverse datasets, CNNs can identify key image features, such as tumors, enhancing accuracy and efficiency in medical diagnostics [14].

The deployment of AI models faces notable hurdles. First, legal and certification concerns arise due to the use of patient data by AI models [15,16]. Second, successful implementation of the model necessitates external validation, including tests on contemporary and geographically diverse patient data, ensuring trustworthiness and generalizability [17,18]. Third, despite the surge in AI model development driven by growing interest, the quality of many papers remains a concern, hindering their transition to clinical practice. The excitement surrounding AI has led to a rapid increase in the number of studies published, yet due to the challenges outlined previously, the majority have not progressed to implementation [18]. Assessing the wide range of AI models focusing on bone metastases to identify those suitable for clinical deployment remains a daunting task, impeding their adoption by healthcare providers.

To address these issues, frameworks such as the Transparent Reporting of a multivariable prediction model for Individual Prognosis Or Diagnosis (TRIPOD) have been established to guide authors in developing AI models and to ensure transparency [19]. The Checklist for Artificial Intelligence in Medical Imaging (CLAIM) has been specifically developed to evaluate AI models that focus on medical imaging [20]. While both tools contribute to establishing and assessing qualitative models, they do not provide a sound recommendation on whether to use the model in clinical practice. The Utility of Prediction Model (UPM) score was developed for this purpose as it assesses AI model performance by scoring AI models on eight items. Which includes sample size, original clinical usability, and other relevant criteria, with scores ranging from 0 to 3 and a maximum total score of 16 [21]. Clinical usefulness and transparency of reporting can be evaluated by combining these three methods, thereby providing honest recommendations on implementation of AI models in daily practice.

Therefore, the aim of this study was to (1) provide an overview of all different AI-modalities in treatment of bone metastases and (2) to provide a recommendation of AI models that are fit for implementation in daily practice based on the TRIPOD, CLAIM and UPM scoring system.

## 2 Methods

### 2.1 Search strategy

This systematic review was conducted adhering to the Preferred Reporting Items for Systematic Reviews and Meta-Analyses (PRISMA) guideline and checklist [22,23] (Appendix 1).

A systematic search was conducted in PubMed, Embase, and the Cochrane Library, encompassing all papers published up to February 29th, 2024.

Three domains of medical subject headings (MeSH) terms and keywords were combined with “AND” and within the two domains the terms were combined with “OR”. The first domain included words related to AI, the second domain to orthopaedic surgery, and the third domain to metastatic bone disease related outcomes. Terms were restricted to MeSH, title, abstract, and keywords (Appendix 2).

### 2.2 Study selection

Studies eligible for inclusion in this systematic review had to utilize AI-based tools, encompassing methodologies such as machine learning (ML) and deep learning (DL). ML was defined as models capable of autonomously learning from data, forming connections, and detecting patterns without human assistance. Additionally, inclusion was limited to studies specifically focusing on bone metastases, which was defined as the presence of metastatic cancer cells in bone tissue (including spinal metastases).

**Fig. 1:**
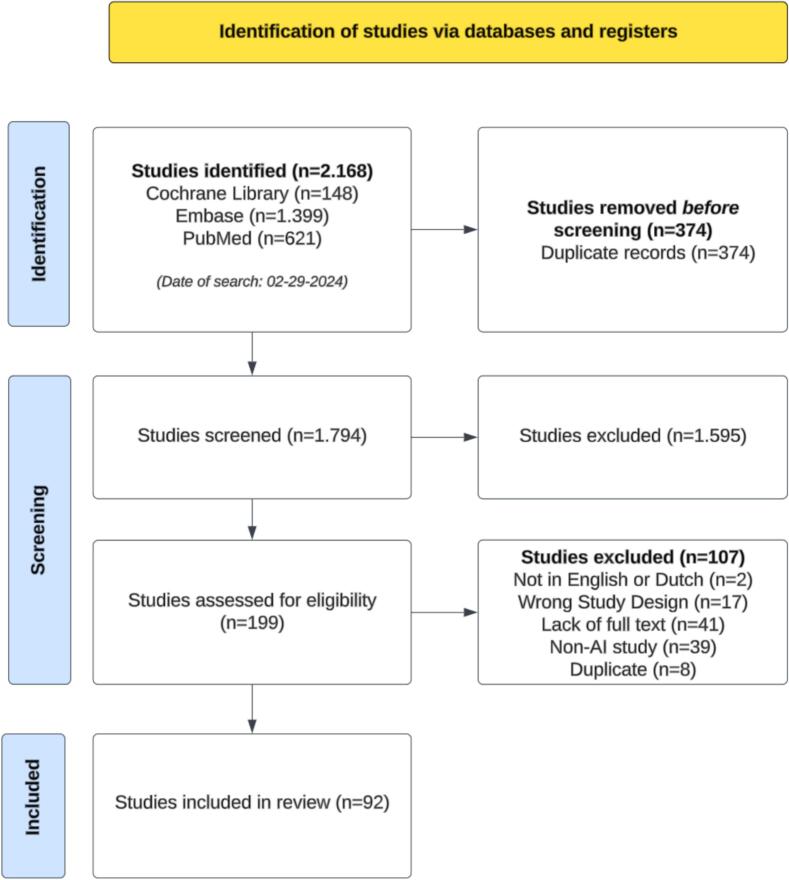
PRISMA flowchart of study inclusions and exclusions * The search was conducted on February 29th 2024.

Exclusion criteria were as follows; 1) non-peer-reviewed publications; 2) studies published in a language other than English or Dutch; 3) papers that did not offer full-text availability; 4) non-relevant study types (animal studies, letters to the editors, case reports, and reviews); 5) studies solely employing non-AI models, such as logistic or linear regression models, were excluded (except for those using AI modalities in conjunction with some form of linear regression) and 6) studies using Natural Language Processing (as there was no tool for accurate assessment such as TRIPOD and CLAIM). In total, 92 studies were included (Fig. 1).

The screening process for this systematic review involved two independent researchers (IV, LRL). The reviewers discussed all discrepancies with two AI-oriented orthopaedic oncology research fellows (TMG, OQG) to reach a consensus.

### 2.3 Quality assessment

All included studies were categorized based on their applications to provide a comprehensive overview of the diverse AI applications: survival prediction, imaging, and others (e.g. studies on the prediction of bone metastases or severe psychological distress).

To give a sound recommendation for AI applications within the treatment of bone metastases, the methodological quality of each included study was assessed through the TRIPOD and the CLAIM. The TRIPOD statement consists of 22 main items testing every prediction model on their quality and transparent reporting of the methods and results. Six items, specifically relating to model updating or external model validation, were exclusively extracted in studies conducting external validation [19]. Items were considered complete when all (sub)items were scored as “yes” (Appendix 3). Completeness was evaluated by dividing the total number of scored ‘’yes’’ (sub)items by the total number of applicable items for developmental, validation, development + validation, and incremental studies.

The CLAIM checklist consists of 44 items, divided into seven sections: title or abstract, abstract, introduction, methods, results, discussion, and other information (Appendix 4). This checklist is specifically designed for evaluating imaging studies and was therefore only assessed for imaging studies [20]. Possible answers included ‘'yes,’' '’no’' or '’not applicable’'. Completeness was calculated based on the percentage of '’yes’' responses for each item.

Lastly, we used the UPM scoring system, wherein studies were rated across 8 domains, including study design, weighted external area under the curve (AUC) and calibration assessment. Weighted external AUC was calculated by multiplying the external validation AUC of each study by its respective sample size and summing these products. The resulting sum was then divided by the total number of patients included across all validation studies. UPM scores were graded into 4 categories: *excellent* (12–16 points), *good* (7–11 points), *fair* (3–6 points), and *poor* (0–2 points) (Table 1) [21].

**Table 1:** UPM scoring system with a score range of 0 – 16.

Predictive model characteristics	Points (0–16)
**Original study sample size**	
<150 patients	0
150–500 patients	1
>500 patients	2
**Original study population**	
Single institution	0
Bi-institutional	1
Multi-institutional	2
**Original study design**	
Retrospective	0
Combined (retro- and prospective)	1
Prospective	2
**Original study AUC**	
<0.70 or not provided	0
0.70–0.80	1
>0.80	2
**Internal validation**	
None	0
Bootstrapping or training/validation	2
**Calibration assessment**	
None	0
Calibration plot/Hosmer-Lemeshow test	2
**Weighted external validation AUC**	
<0.70 or no external validation	0
0.70–0.80	2
>0.80	3
**Clinical usability**	
No WBC	0
WBC	1

AUC = area under the curve, WBC = web-based calculator, scoring system: excellent = 12–16 points, good = 7–11 points, fair = 3–6 points, poor = 0–2 points.

To demonstrate the clinical usefulness of AI modalities, a cutoff score was established. When the completeness of TRIPOD or CLAIM was equal to or greater than 70 % and the UPM score was equal to or greater than 10, a study was labeled as clinically useful. This cutoff score was established after careful consideration of the current literature and assessment of our own data [[24], [25], [26], [27]]. A particular emphasis on several crucial aspects in evaluating a model's value, such as handling missing data, calibration, and discrimination metrics was made [[28], [29], [30]].

When studies developed multiple AI models only the best-performing model was assessed for evaluation of performance metrics (AUC). Furthermore, when multiple internal and/or (weighted) external AUC were presented, we documented all AUCs for the different time points and calculated their mean, which was used for calculating the UPM score.

Using both the TRIPPOD and CLAIM ensures an appropriate assessment of the quality of each type of study. We used the TRIPOD statement for prediction models (prognostic models), validation studies (studies aimed at validating/evaluating existing model(s)), and 'other' studies, while we employed the CLAIM for imaging studies. Additionally, to assess clinical usefulness, all developmental studies were scored following the UPM scoring system.

### 2.4 Statistical analysis

The degree of completeness of reporting to TRIPOD statement and the CLAIM checklist domains were calculated and presented with percentages using medians with interquartile ranges (IQRs). All visualization methods, including graphs and pie charts, were created using Excel version 16.81 (Microsoft Corp, Redmond, WA, USA). Statistical analysis was conducted using SPSS version 28 (IBM, USA).

## 3 Results

### 3.1 Included studies

A total of 2.168 studies were initially identified, and after removing duplicates, 1.794 studies remained (Fig. 1). Title and abstract screening resulted in 199 potentially relevant studies. After full-text review, 107 studies were excluded due to various reasons, including incorrect study design (n = 17), absence of full text (n = 41), non-AI focus (n = 39), being in a language other than English or Dutch (n = 2), and the presence of duplicates (n = 8). In total, 92 studies were included.

### 3.2 Study characteristics

**Fig. 2:**
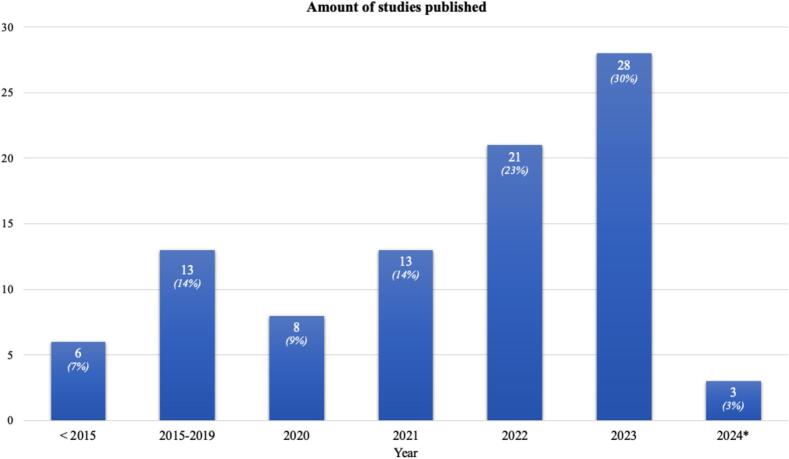
Overview of growing amount of studies published on AI in bone metastases.

**Fig. 3:**
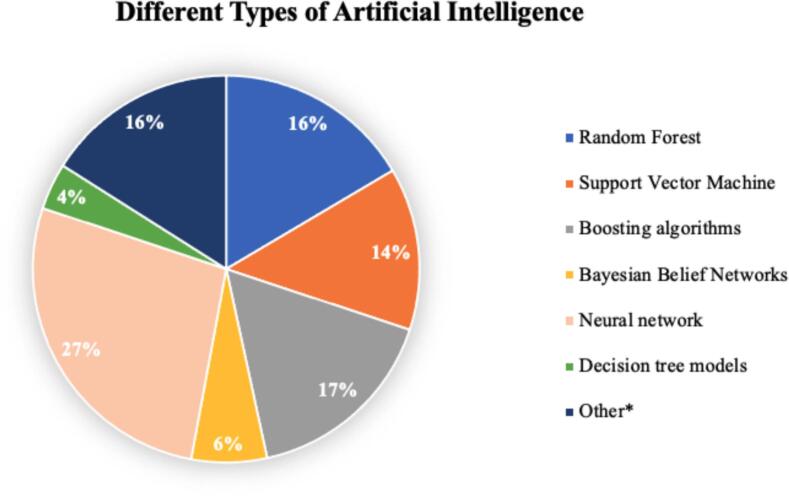
Overview of used AI techniques in all 92 models. * Other refers to: Elastic Net Penalized Logistic Regression (48%; 16/33), Logistic Regression (15%; 5/33), Lasso Regularization Logistic Regression (6%; 2/33), Computer aided detection (3%; 1/33), OG-Domain Diffeomorphic Demons Algorithm (3%; 1/33), Multi View-Attention-Guided Network (3% 1/33), Bayes Point Machine (3%; 1/33), Naive Bayes (3%; 1/33), Ensemble Prediction (3%; 1/33), Masked Thresholding (3%; 1/33), Radiomics (3%; 1/33), CatBoost Classifier (3%; 1/33), and Bayesian Classifier (3%; 1/33).

Most studies were published in 2023 (28/92; 30 %), followed by 2022 (21/92; 23 %) (Fig. 2). Most studies (75/92; 82 %) used electronical health records as their database, while 17 studies (17/92; 18 %) relied on registries (Table 2). Most included studies consisted of developmental studies (49/92; 53 %) followed by validation studies (33/92; 36 %), combination of development and validation (8/92; 9 %) and other (2/92; 2 %, including various types of logistic regression and neural networks). Among the 92 included studies, a total of 206 AI modalities were used. The most frequently reported types of AI were neural networks (56/206; 27 %), random forest and boosting algorithms (34/206; 17 %). The least used type of AI was bayesian belief network (13/206; 6 %) and decision tree models (8/206; 4 %) (Fig. 3). Most studies focused primarily on prognostication (survival prediction) (44/92;48 %), followed by imaging (bone metastases diagnosis, object detection and lesion segmentation) studies (37/92; 40 %) and other (11/92; 12 %). Each of the three categories is described in detail below, using the three quality assessment tools.

**Table 2:** Characteristics of all included studies (n = 92).

Variables	Values
Median patient sample size (IQR)	399 (189 to 1.043)
Year of publication, n (%)	
2024 “February 29th”	3 (3)
2023	28 (30)
2022	21 (23)
2021	13 (14)
2020	8 (9)
2015 – 2019	13 (14)
<2015	6 (7)
Type of database used, n (%)	
Electronic health record	75 (82)
Registry	17 (18)
Type of paper, n (%)	
Development	49 (53)
Validation	33 (36)
Combination	8 (9)
Other	2 (2)
Location, n (%)	
Spine	37 (40)
Appendicular skeleton	13 (14)
Combined	42 (46)
Outcome, n (%)	
Survival	46 (50)
Detection	28 (30)
Segmentation	5 (5)
Prediction	4 (4)
Other	9 (10)
Type of algorithm used, n (%)	
Neural Network (NN)	56 (27)
Random Forest (RF)	34 (17)
Boosting algorithms	34 (17)
Other*	33 (16)
Support Vector Machine (SVM)	28 (14)
Bayesian Belief Network (BBN)	13 (6)
Decision Tree models (DT)	8 (4)

IQR = interquartile range.

* Other refers to: Elastic Net Penalized Logistic Regression (48%; 16/33), Logistic Regression (15%; 5/33), Lasso Regularization Logistic Regression (6%; 2/33), Computer aided detection (3%; 1/33), OG-Domain Diffeomorphic Demons Algorithm (3%; 1/33), Multi View-Attention-Guided Network (3% 1/33), Bayes Point Machine (3%; 1/33), Naive Bayes (3%; 1/33), Ensemble Prediction (3%; 1/33), Masked Thresholding (3%; 1/33), Radiomics (3%; 1/33), CatBoost Classifier (3%; 1/33), and Bayesian Classifier (3%; 1/33).

### 3.3 Survival prediction

Forty-four (48 %) studies focused on survival prediction, comprising 18 (41 %) developmental studies and 26 (59 %) validation studies, with most of the studies published in 2022 (12/44; 27 %). The median sample size was 412 (IQR 189 to 1070) patients, and the median internal AUC (development studies) was 0.81 (IQR 0.78 to 0.84). Almost half of the developmental studies (8/18; 44 %) provided a web-based calculator or published their development code online. The most used type of AI in the developmental studies was boosting algorithms (14/56; 25 %), closely followed by Random Forest (11/52; 20 %). Among the 18 developmental studies on survival prediction, 6 (33 %) were fit for clinical use (Table 3).

**Table 3:** AI models fit for clinical use (n = 9).

First Author	Study type	Year of publication	Outcome	TRIPOD completeness (%)	CLAIM completeness (%)	UPM Score
Survival prediction						
Forsberg, JA [46]	D	2017	Survival	75 %	−	10 (Good)
Karhade, AV [3]	D + V	2022	Survival	85 %	−	13 (Excellent)
Karhade, AV [4]	D	2019	Survival	73 %	−	12 (Excellent)
Karhade, AV [47]	D	2022	Survival	75 %	−	10 (Good)
Le, Y [48]	D	2023	Survival	70 %	−	11 (Good)
Thio, QCBS [7]	D	2020	Survival	73 %	−	11 (Good)
Imaging, detection, and segmentation						
Hallinan, JTPD [49]	D + V	2022	Detection	−	74 %	10 (Good)
Liu, X [39]	D	2021	Detection	−	83 %	11 (Good)
Xiong, Y [41]	D	2023	Detection	−	79 %	11 (Good)

AI modalities are rated as ‘fit for clinical use’ when TRIPOD or CLAIM completeness was ≥ 70 %, and UPM score ≥ 10.

Abbreviations: TRIPOD = Transparent Reporting of a multivariable prediction model for Individual Prognosis Or Diagnosis; CLAIM= Checklist for Artificial Intelligence in Medical Imaging; UPM = Utility of Prediction Model score; D = developmental study; V = external validation study; D + V = development and external validation

Overall TRIPOD median completeness for all 44 survival prediction models was 73 % (IQR 67 % to 84 %) (Table 4). The highest TRIPOD completeness of 94 % was achieved in the validation study conducted by Bongers et al.[31], whereas the highest completeness for developmental studies was 93 % by Janssen et al. [12].

**Table 4:** Completeness > 90 % and < 25 % of TRIPOD for all included survival prediction and ‘other’ studies (n = 55).

TRIPOD item	TRIPOD % (n/total)
Complete reporting > 90 %	
3b − Specify the objectives, including whether the study describes the development or validation of the model or both.	93 (51/55)
4a − Describe the study design or source of data (e.g., randomized trial, cohort, orregistry data) , separately for the development and validation data sets, if applicable	95 (52/55)
18 − Discuss any limitations of the study (such as nonrepresentative sample, few events per predictor, missing data).	96 (53/55)
19a − Give an overall interpretation of the results, considering objectives, limitations, and results from similar studies, and other relevant evidence.	97 (31/32) *
19b − Give an overall interpretation of the results, considering objectives, limitations, and results from similar studies, and other relevant evidence.	96 (52/55)
Complete reporting < 25 %	
10e − Describe any model updating (e.g., recalibration) arising from the validation, if done	19 (6/32) *
*TRIPOD = Transparent Reporting of a multivariable prediction model for Individual Prognosis Or Diagnosis*	

*External validation was performed in 30 studies and 2 studies were found to be an incremental study, resulting in a total of 32 studies for items 10e and.

UPM scores ranged from 5 to 13 with a median of 10 (IQR 7 to 11). The model by Karhade et al. achieved the highest UPM score (13 points) in predicting six-week mortality in spinal metastatic disease (n = 4.304), (Appendix A, [33], [34], [35], [36], [37], [38], [50], [51], [52], [53], [54], [55], [56], [57], [58], [59], [60], [61], [62], [63], [64], [65], [66], [67], [68], [69], [70], [71], [72], [73], [74], [75], [76], [77], [78], [80], [81], [82], [83], [84], [85], [86], [87], [88], [89], [90], [91], [92], [93], [94], [95], [96], [97], [98], [99], [100], [101], [102], [103], [104], [105], [79], [106], [107], [108], [109], [110], [111], [112], [113], [114], [115], [116], [117]) [3]. The model used multi-institutional data and was constructed using penalized logistic regression, categorized as an 'other' type of AI, and demonstrated an internal AUC of 0.84 and an external AUC of 0.81 [3]. Upon external validation using 2.768 patients the model showed a mean external AUC of 0.78 [32]. The second-best model based on UPM score (12 points) was also by Karhade et al. evaluating 90-day and 1 year mortality in patients with spinal metastases (n = 732) using boosting algorithms to establish the model. Its performance yielded an internal mean AUC of 0.84 and with external validation the model demonstrated a weighted external AUC of 0.80 [6,[10], [11], [12], [13], [14],^15^]. Thio et al. also developed a”stochastic gradient boosting” model with an UPM score of 12 points for preoperative survival prediction of extremity metastatic disease with a TRIPOD completeness of 84 % [7]***.***

### 3.4 Bone metastases, object detection and lesion segmentation

Among 37 studies on bone metastases diagnosis, object detection, lesion segmentation, the median sample size was 269 (IQR 116 to 675) patients and most studies were published in 2023 (16/37; 43 %). The majority of the studies (24/37; 65 %), presented an internal AUC with a median of 0.88 (IQR 0.82 to 0.96). Three studies (3/37; 8 %) provided a web-based calculator or published their development code online. Neural Network was the most used type of AI (32/39; 82 %). Most studies focused on object detection (28/77; 76 %), followed by lesion segmentation (5/37; 14 %) and prediction (4/37; 11 %). Three out of the 37 studies (8 %) are labeled as fit for clinical use (Table 3).

CLAIM completeness was assessed for all studies, ranging from 31 % to 83 % with a median completeness of 57 % (IQR 50 % to 70 %) (Table 5). Item 19 (intended sample size and how it was determined) showed the lowest completeness (0 %), while item 2–6 (focused on title/abstract, introduction, and study design) showed 100 % completeness. The study by Liu et al., demonstrated the highest overall completeness with 83 % [39], followed by Hallinan et al. and Xiong et al., both presenting an overall completeness of 79 % (S upplementary Table S3) [40,41].

**Table 5:** Completeness > 90 % and < 25 % of CLAIM for all included imaging studies (n = 37).

CLAIM item	CLAIM % (n/total)
Complete reporting > 90 %	
1 – Identification as a study of AI methodology, specifying the category of technology used (e.g., deep learning)	95 (35/37)
2 – Structured summary of study design, methods, results, and conclusions	100 (37/37)
3 – Scientific and clinical background, including the intended use and clinical role of the AI approach	100 (37/37)
4 – Study objectives and hypotheses	100 (37/37)
5 – Prospective or retrospective study	92 (34/37)
6 – Study goal, such as model creation, exploratory study, feasibility study, non-inferiority trial	100 (37/37)
7 – Data sources	97 (36/37)
28 – Metrics of model performance	92 (34/37)
35 – Performance metrics for optimal model(s) on all data partitions	95 (35/37)
39 – Implications for practice, including the intended use and/or clinical role	100 (37/37)
Complete reporting < 25 %	
12 – De-identification methods	8 (3/37)
13 – How missing data were handled	14 (5/37)
18 – Measurement of inter- and intrarater variability; methods to mitigate variability and/or resolve discrepancies	16 (6/37)
19 – Intended sample size and how it was determined	0 (0/37)
30 – Robustness or sensitivity analysis	14 (5/37)
*CLAIM= Checklist for Artificial Intelligence in Medical Imaging; AI = Artificial Intelligence*	

The UPM score ranged from 2 to 11, with a median score of 5 (IQR 4 to 7) (S upplementary Table S3). Xiong et al. developed a deep learning model to differentiate bone islands from osteoblastic bone metastases using CT images, achieving the highest UPM score of 11 (n = 728). The study showed an internal AUC of 0.99 and external AUCs of 0.96 and 0.95 [41]. Lui et al. assessed the detection and segmentation of pelvic bone metastases in MRI images for patients with prostate cancer and achieved a UPM score of 11, indicating good clinical utility [39].

### 3.5 Other models

Eleven studies were categorized as ‘other’, mainly because they focused on less-studied outcomes such as psychological distress and pain reduction. Most studies were published in 2021 (4/11; 36 %). The median sample size across these studies was 1.143 (IQR 534 to 12591) patients, with a median internal AUC of 0.88 (IQR 0.83 to 0.92). Many studies (6/11; 55 %) offered accessibility through a web-based calculator or by publishing their development code online. Random forest was the most frequent type of AI (7/28; 25 %), followed by boosting algorithms (6/28; 21 %) and support vector machine (5/28; 18 %). None were deemed fit for clinical use. Median TRIPOD score completeness for all other categorized studies was 53 % (IQR 49 % to 64 %). Huang et al. showed highest overall TRIPOD completeness with 89 % [42]. The UPM score spanned from 4 to 9, with a median score of 8 (IQR 6 to 9). Four studies achieved a UPM score of 9 [[42], [43], [44], [45]] (S upplementary Table S4).

## 4 Discussion

This study provided a current overview of the 92 AI models published in the treatment of metastatic bone disease and provides a recommendation of AI models that are fit for implementation in daily practice based on three established tools. By thoroughly evaluating all related studies, we identified limitations in the current literature, thereby shedding light on areas for future development and validation of AI models.

### 4.1 Clinical usability of AI models

Remarkably, only nine AI modalities met our criteria for clinical usability including six for survival prediction [3,4,7,[46], [47], [48]] and three for imaging [39,41,49]. This low number of clinically usable AI models may be attributed to the established cut-off score. Lowering the cut-off score results in more qualified studies. However, setting high standards will only recommend the best-performing models and will eventually lead to overall better-performing models. Developing good and reliable models is mandatory to gain the trust and adoption of both patients and clinicians in daily practice.

It is important to note which items contribute to achieving the 70 % TRIPOD cut-off score, as the value of an AI study is primarily determined by its methods and results sections, particularly the presence of all necessary performance metrics such as AUCs, calibration curves, and decision curve analysis. All six studies which were found ‘fit for clinical use’ have 100 % TRIPOD completeness on the above-mentioned criteria, underscoring its importance. This principle extends to the CLAIM checklist as well. It is vital to bear in mind that studies labeled as non-clinically useful are not necessarily inferior; these studies may require more model updating and external validation before they can be implemented into daily practice.

Recommending AI models that are fit for clinical use establishes a clear statement amidst the vast array of AI studies. There has been a notable surge in the number of studies published on AI in bone metastases over the years. This can be attributed to the widespread hype surrounding AI technologies. As observed in our analysis, a small sample of published AI models exhibits low adherence to appropriate guidelines and demonstrates poor clinical usability. Hopefully, this systematic review will contribute to more transparent reporting, increase the number of qualitative studies, and lead to the possible implementation of AI modalities in metastatic bone disease.

### 4.2 Challenges in quality assessment tools

The TRIPOD statement, published in 2015, is very extensive and thorough [19]. However, despite its advantages, it also has its shortcomings. To assess the completeness of items, all (sub)items need to be scored complete. Several items have multiple subitems, making it difficult to achieve completeness. For example, item 2 has 12 subitems. This challenge is also observed with the CLAIM checklist [20]. Furthermore, for both the TRIPOD and the CLAIM the relative significance of each item and the criteria for defining an acceptable score are yet unknown and open to debate. Additionally, because of the extensive nature of the TRIPOD statement, it might not always be useful or suitable for authors to document all items. Developing an updated TRIPOD statement that incorporates all the points may potentially lead to increased utilization by authors and greater overall completeness of the TRIPOD statement. Moreover, adherence to TRIPOD or CLAIM guidelines is not currently required when submitting manuscripts to journals. Our results clearly indicate that there is room for improvement in the completeness of TRIPOD and CLAIM guidelines. Therefore, journals should consider incorporating guidelines such as TRIPOD and CLAIM in their review process to enhance the quality of published AI studies. In our study, 20 out of 55 studies (36 %) adhered to the TRIPOD guideline, while the majority did not (35/55; 64 %). Studies that adhered to the TRIPOD statement demonstrated a median completeness of 79 % (IQR 73 % to 85 %), whereas those that did not adhere to the TRIPOD statement had a lower overall completeness of 68 % (IQR 60 % to 73 %). This finding illustrates that studies adhering to the TRIPOD guideline achieved higher overall completeness (p = 0.001), emphasizing the importance of appropriate guideline adherence.

### 4.3 Limitations

This study has several limitations. Firstly, the UPM score has broad applicability and is easy to use, but the evaluation of data-related aspects, such as the year of inclusion of the data cohort and the year of study publication, are aspects that represent crucial elements in determining the value of a model in current times. The UPM score as well as the CLAIM are lacking this information, which could lead to imperfect assessments. The TRIPOD guides authors in developing qualitative AI models and manuscripts; therefore, data-related aspects are not applicable to the TRIPOD. Secondly, the UPM score fails to address the issue of missing data. This potentially leads to a biased predictor outcome due to an unrepresentative study population. Thirdly, the UPM score exclusively assesses the AUC and does not consider other performance metrics like calibration slope, calibration intercept, Brier score, or decision curve analysis (DCA). This presents a considerable limitation as it may lead to an incomplete depiction of the model’s performance. While the UPM score includes an item for 'calibration assessed', incorporating multiple performance metrics rather than solely relying on the AUC would provide a more comprehensive representation of the model’s performance. Fourthly, the UPM score is only applicable to developmental studies, due to establishment of the scoring system, making it unsuitable for validation studies. Validation studies are unable to demonstrate internal AUCs or weighted external AUCs, which leads to a biased assessment. Lastly, the absence of a web-based calculator – which is a factor in the UPM score – does not diminish a model’s quality or clinical usefulness; rather, providing a calculator improves accessibility and encourages external validation.

Despite all limitations, this study provides the first comprehensive overview of the quality of AI-based models in bone metastases using both the TRIPOD, CLAIM and UPM score. It is vital to promote clear, transparent, and reproducible scientific communication about the application of AI in healthcare. Illustrating poor reporting of certain TRIPOD and CLAIM items highlighted in this systematic review identifies current shortcomings and may improve transparent reporting in the future. In addition, it is essential to prioritize next steps toward implementation, which include worldwide collaboration on how AI models handle, store, and process patient data.

### 4.4 Future directions for the clinical implementation of AI modalities

AI models face several challenges in development and clinical implementation, including generalizability, model aging, legal and ethical concerns, and the need for interdisciplinary collaboration [15,16]. Model generalizability remains a key issue, as external validation across diverse populations is essential. While global models offer broad applicability, they often sacrifice performance compared to region-specific models. Future AI should focus on adaptive learning methods, such as federated learning, to enhance generalizability without compromising accuracy [17,18].

Model aging, where older models become outdated as medical science advances is seen as another challenge [2]. Continuous learning algorithms that integrate real-time clinical data are crucial for maintaining model relevance. Developing self-updating AI frameworks will help models evolve with changing healthcare practices.

Legal and ethical considerations also hinder AI adoption, as models must comply with country-specific regulations on patient data privacy and security. Global deployment adds complexity due to varying legal frameworks. Future efforts should focus on developing standardized international guidelines to facilitate AI integration while ensuring compliance with ethical and legal standards [14].

Technological advancements and interdisciplinary collaboration will play a crucial role in AI’s future. Integrating AI with imaging modalities, electronic health records, and multi-omics data can improve diagnostic accuracy. Collaboration between AI developers, clinicians, and policymakers will be essential to refine algorithms and ensure real-world applicability.

To address these challenges, AI development should prioritize robust validation frameworks, continuous learning mechanisms, ethical data policies, and cross-disciplinary collaboration. By implementing these strategies, AI can become a clinically effective and ethically responsible tool, improving patient outcomes and fostering broader clinical adoption.

## 5 Conclusion

In conclusion, only nine out of the 92 currently available AI models in bone metastases were deemed fit for clinical use. The low number of clinically useful models illustrates the need for better adherence to established guidelines to ensure transparency and quality. Especially more focus on the methods and results ensures reliable and accurate models. Both patients and clinicians will take advantage of only clinically useful AI models which may improve AI-driven personalized cancer treatment.

## CRediT authorship contribution statement

**Lotte R. van der Linden:** Writing – review & editing, Writing – original draft, Project administration, Methodology, Formal analysis, Data curation. **Ioannis Vavliakis:** Methodology, Investigation. **Tom M. de Groot:** Writing – review & editing, Methodology, Investigation, Conceptualization. **Paul C. Jutte:** Writing – review & editing, Supervision. **Job N. Doornberg:** Writing – review & editing, Supervision. **Santiago A. Lozano-Calderon:** Writing – review & editing, Supervision. **Olivier Q. Groot:** Writing – review & editing, Supervision, Methodology, Conceptualization.

## Declaration of competing interest

The authors declare that they have no known competing financial interests or personal relationships that could have appeared to influence the work reported in this paper.
