# Supplementary material for: Artificial Intelligence in bone Metastases: A systematic review in guideline adherence of 92 studies
Source: J Bone Oncol. 2025 Apr 24;52:100682. doi: 10.1016/j.jbo.2025.100682 (PMC12056386; doi:10.1016/j.jbo.2025.100682)
Supplement: Supplementary Data 1 [file mmc1.docx]

**Table 1.** UPM scoring system with a score range of 0 – 16.

| Predictive model characteristics | Points (0-16) |
| --- | --- |
| **Original study sample size** |  |
| <150 patients | 0 |
| 150–500 patients | 1 |
| >500 patients | 2 |
| **Original study population** |  |
| Single institution | 0 |
| Bi-institutional | 1 |
| Multi-institutional | 2 |
| **Original study design** |  |
| Retrospective | 0 |
| Combined (retro- and prospective) | 1 |
| Prospective | 2 |
| **Original study AUC** |  |
| <0.70 or not provided | 0 |
| 0.70–0.80 | 1 |
| >0.80 | 2 |
| **Internal validation** |  |
| None | 0 |
| Bootstrapping or training/validation | 2 |
| **Calibration assessment** |  |
| None | 0 |
| Calibration plot/Hosmer-Lemeshow test | 2 |
| **Weighted external validation AUC** |  |
| <0.70 or no external validation | 0 |
| 0.70–0.80 | 2 |
| >0.80 | 3 |
| **Clinical usability** |  |
| No WBC | 0 |
| WBC | 1 |
| *AUC = area under the curve,* *WBC = web-based calculator, scoring system: excellent = 12–16 points, good = 7–11 points, fair = 3–6 points, poor= 0–2 points.* | |

**Table 2**. Characteristics of all included studies (n=92)

**Variables Values**

| Median patient sample size (IQR) | 399 (189 to 1.043) |
| --- | --- |
| Year of publication, n (%) |  |
| 2024 “February 29^th^” | 3 (3) |
| 2023 | 28 (30) |
| 2022 | 21 (23) |
| 2021 | 13 (14) |
| 2020 | 8 (9) |
| 2015 – 2019 | 13 (14) |
| <2015 | 6 (7) |
| Type of database used, n (%) |  |
| Electronic health record | 75 (82) |
| Registry | 17 (18) |
| Type of paper, n (%) |  |
| Development | 49 (53) |
| Validation | 33 (36) |
| Combination | 8 (9) |
| Other | 2 (2) |
| Location, n (%) |  |
| Spine | 37 (40) |
| Appendicular skeleton | 13 (14) |
| Combined | 42 (46) |
| Outcome, n (%) |  |
| Survival | 46 (50) |
| Detection | 28 (30) |
| Segmentation | 5 (5) |
| Prediction | 4 (4) |
| Other | 9 (10) |
| Type of algorithm used, n (%) |  |
| Neural Network (NN) | 56 (27) |
| Random Forest (RF) | 34 (17) |
| Boosting algorithms | 34 (17) |
| Other* | 33 (16) |
| Support Vector Machine (SVM) | 28 (14) |
| Bayesian Belief Network (BBN) | 13 (6) |
| Decision Tree models (DT) | 8 (4) |
| *IQR= interquartile range*  ** Other refers to: Elastic Net Penalized Logistic Regression (48%; 16/33), Logistic Regression (15%; 5/33), Lasso Regularization Logistic Regression (6%; 2/33), Computer aided detection (3%; 1/33), OG-Domain Diffeomorphic Demons Algorithm (3%; 1/33), Multi View-Attention-Guided Network (3% 1/33), Bayes Point Machine (3%; 1/33), Naive Bayes (3%; 1/33), Ensemble Prediction (3%; 1/33), Masked Thresholding (3%; 1/33), Radiomics (3%; 1/33), CatBoost Classifier (3%; 1/33), and Bayesian Classifier (3%; 1/33).* | |

| First Author | Study type | Year of publication | Outcome | TRIPOD completeness  (%) | CLAIM completeness (%) | UPM Score |
| --- | --- | --- | --- | --- | --- | --- |
| Survival prediction |  |  |  |  |  |  |
| Forsberg, JA [46] | D | 2017 | Survival | 75% | - | 10 (Good) |
| Karhade, AV [3] | D+V | 2022 | Survival | 85% | - | 13 (Excellent) |
| Karhade, AV [4] | D | 2019 | Survival | 73% | - | 12 (Excellent) |
| Karhade, AV [47] | D | 2022 | Survival | 75% | - | 10 (Good) |
| Le, Y [48] | D | 2023 | Survival | 70% | - | 11 (Good) |
| Thio, QCBS [7] | D | 2020 | Survival | 73% | - | 11 (Good) |
| Imaging, detection, and segmentation |  |  |  |  |  |  |
| Hallinan, JTPD [49] | D+V | 2022 | Detection | - | 74% | 10 (Good) |
| Liu, X [39] | D | 2021 | Detection | - | 83% | 11 (Good) |
| Xiong, Y [41] | D | 2023 | Detection | - | 79% | 11 (Good) |

**Table 3.** AI models fit for clinical use (n=9).

*AI modalities are rated as ‘fit for clinical use’ when TRIPOD or CLAIM completeness was ≥ 70%, and UPM score ≥ 10.*

*Abbreviations: TRIPOD= Transparent Reporting of a multivariable prediction model for Individual Prognosis Or Diagnosis; CLAIM=* *Checklist for Artificial Intelligence in Medical Imaging; UPM= Utility of Prediction Model score; D= developmental study; V=external validation study; D+V= development and external validation*

**Table 4**. Completeness >90% and <25% of TRIPOD for all included survival prediction and ‘other’ studies (n=55).

**TRIPOD item TRIPOD % (n/total)**

| Complete reporting >90% |  |
| --- | --- |
| 3b - Specify the objectives, including whether the study describes the development or validation of the model or both. | 93 (51/55) |
| 4a - Describe the study design or source of data (e.g., randomized trial, cohort, or  registry data), separately for the development and validation data sets, if applicable | 95 (52/55) |
| 18 - Discuss any limitations of the study (such as nonrepresentative sample, few events per predictor, missing data). | 96 (53/55) |
| 19a - Give an overall interpretation of the results, considering objectives, limitations, and results from similar studies, and other relevant evidence. | 97 (31/32) * |
| 19b - Give an overall interpretation of the results, considering objectives, limitations, and results from similar studies, and other relevant evidence. | 96 (52/55) |
| Complete reporting <25% |  |
| 10e - Describe any model updating (e.g., recalibration) arising from the validation, if done | 19 (6/32) * |
| *TRIPOD= Transparent Reporting of a multivariable prediction model for Individual Prognosis Or Diagnosis* | |

**External validation was performed in 30 studies and 2 studies were found to be an incremental study, resulting in a total of 32 studies for items 10e and*

**Table 5**. Completeness >90% and <25% of CLAIM for all included imaging studies (n=37).

**CLAIM item CLAIM % (n/total)**

| Complete reporting >90% |  |
| --- | --- |
| 1 – Identification as a study of AI methodology, specifying the category of technology used (e.g., deep learning) | 95 (35/37) |
| 2 – Structured summary of study design, methods, results, and conclusions | 100 (37/37) |
| 3 – Scientific and clinical background, including the intended use and clinical role of the AI approach | 100 (37/37) |
| 4 – Study objectives and hypotheses | 100 (37/37) |
| 5 – Prospective or retrospective study | 92 (34/37) |
| 6 – Study goal, such as model creation, exploratory study, feasibility study, non-inferiority trial | 100 (37/37) |
| 7 – Data sources | 97 (36/37) |
| 28 – Metrics of model performance | 92 (34/37) |
| 35 – Performance metrics for optimal model(s) on all data partitions | 95 (35/37) |
| 39 – Implications for practice, including the intended use and/or clinical role | 100 (37/37) |
| Complete reporting <25% |  |
| 12 – De-identification methods | 8 (3/37) |
| 13 – How missing data were handled | 14 (5/37) |
| 18 – Measurement of inter- and intrarater variability; methods to mitigate variability and/or resolve discrepancies | 16 (6/37) |
| 19 – Intended sample size and how it was determined | 0 (0/37) |
| 30 – Robustness or sensitivity analysis | 14 (5/37) |
| *CLAIM= Checklist for Artificial Intelligence in Medical Imaging; AI= Artificial Intelligence* | |

**
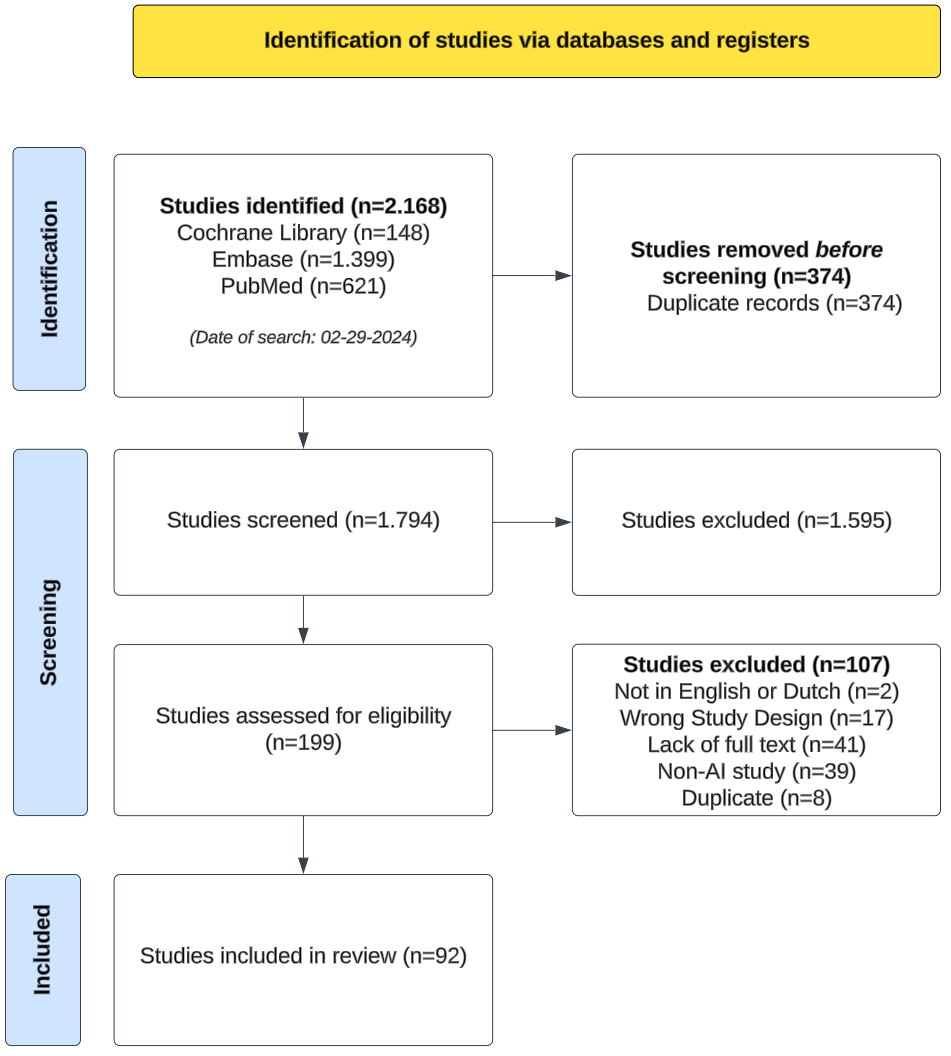
**

**Figure 1.** PRISMA flowchart of study inclusions and exclusions


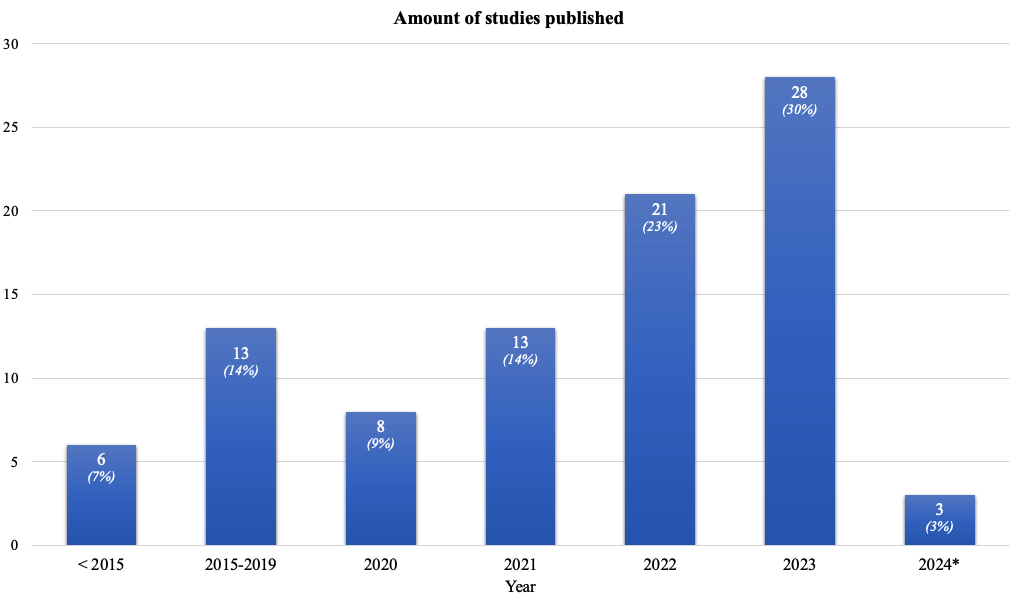


** The search was conducted on February 29^th^ 2024.*

**Figure 2.** Overview of growing amount of studies published on AI in bone metastases.


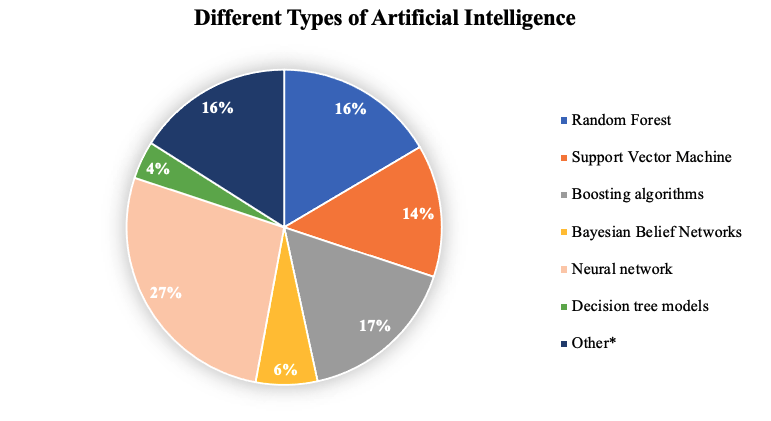


**Figure 3.** Overview of used AI techniques in all 92 models. ** Other refers to: Elastic Net Penalized Logistic Regression (48%; 16/33), Logistic Regression (15%; 5/33), Lasso Regularization Logistic Regression (6%; 2/33), Computer aided detection (3%; 1/33), OG-Domain Diffeomorphic Demons Algorithm (3%; 1/33), Multi View-Attention-Guided Network (3% 1/33), Bayes Point Machine (3%; 1/33), Naive Bayes (3%; 1/33), Ensemble Prediction (3%; 1/33), Masked Thresholding (3%; 1/33), Radiomics (3%; 1/33), CatBoost Classifier (3%; 1/33), and Bayesian Classifier (3%; 1/33).*

**Supplementary table 1**. Overview of the included studies (n=92)

| First author | Year of publication | Journal | PMID |
| --- | --- | --- | --- |
| Ahmed, AK [50] | 2018 | The Spine Journal | 29567516 |
| Albaradei, S [43] | 2021 | Frontiers in Genetics | 34858485 |
| Alcorn, SR [51] | 2020 | International Journal of Radiation Oncology, Biology, Physics | 32446952 |
| Alfaro, PA [52] | 2021 | European Journal of Orthopedic Surgery & Traumatology | 34677661 |
| Anderson, AB [53] | 2022 | BMC Cancer | 35490227 |
| Anderson, AB [1] | 2020 | Clinical Orthopedic Related Research | 32195761 |
| Bongers, ME [31] | 2020 | The Spine Journal | 32428674 |
| Cai, WL [54] | 2024 | Computers in Biology and Medicine | 38000245 |
| Carrwik, C [55] | 2022 | Brain & Spine Journal | 36506283 |
| Ceranka, J [56] | 2023 | Computer Methods and Programs in Biomedicine | 37742486 |
| Chang, CY [57] | 2022 | Skeletal Radiology | 34291325 |
| Chavalparit, P [58] | 2023 | Asian Spine Journal | 38050361 |
| Chen, K [59] | 2022 | Frontiers in Oncology | 36158659 |
| Chen, YY [60] | 2023 | Diagnostics | 37835785 |
| Chiu, JS [61] | 2009 | Journal of Medical Systems | 19397093 |
| Chmelik, J [62] | 2018 | Medical Image Analysis | 30114549 |
| Cui, Y [63] | 2022 | Frontiers in Public Health | 36276398 |
| Cui, Y [64] | 2022 | Frontiers in Oncology | 36568149 |
| de Groot, TM [2] | 2023 | Clinical Orthopedic Related Research | 37229565 |
| Duan, S [65] | 2023 | Infection and Drug Resistance | 37424672 |
| Elledge, CR [66] | 2021 | JCO Clinical Cancer Informatics | 33760638 |
| Forsberg, JA [67] | 2011 | PLoS One | 21603644 |
| Forsberg, JA [68] | 2012 | BMC Cancer | 23098538 |
| Forsberg, JA [69] | 2013 | Clinical Orthopedic Related Research | 22983682 |
| Forsberg, JA [46] | 2017 | Clinical Orthopedic Related Research | 27909972 |
| Gao, L [44] | 2023 | The Spine Journal | 37182703 |
| Gui, C [70] | 2021 | Journal of Neurosurgery | 34560656 |
| Hallinan, JTPD [49] | 2022 | Frontiers in Oncology | 37213273 |
| Hallinan, JTPD [40] | 2023 | Frontiers in Oncology | 35600347 |
| Hallinan, JTPD [71] | 2022 | Cancers | 35804990 |
| Hammon, M [72] | 2013 | European Radiology | 23397381 |
| Hoshiai, S [73] | 2022 | European Journal of Radiology | 35901601 |
| Hsieh, HC [74] | 2022 | Acta Orthopaedica | 36083697 |
| Hsieh, T [75] | 2021 | Journal of Personalized Medicine | 34945720 |
| Huang, CC [42] | 2023 | Clinical Orthopaedics and Related Research | 37306629 |
| Huang, Z [76] | 2020 | BioMed Research International | 32685470 |
| Huo, T [77] | 2023 | Frontiers in Oncology | 36845701 |
| Ibrahim, A [78] | 2023 | Cancer Imaging | 36698217 |
| Janssen, SJ [12] | 2015 | Clinical Orthopaedics and Related Research | 26155769 |
| Karhade, AV [33] | 2020 | The Spine Journal | 31505303 |
| Karhade, AV [79] | 2022 | The Spine Journal | 35843533 |
| Karhade, AV [4] | 2019 | Neurosurgery | 30869143 |
| Karhade, AV [47] | 2022 | Neurosurgery | 30476188 |
| Kikushima, S [80] | 2015 | Annals of Nuclear Medicine | 25348548 |
| Koike, Y [81] | 2023 | International Journal of Computer Assisted Radiology and Surgery | 36991276 |
| Lang, N [82] | 2019 | Magnetic Resonance Imaging | 30826448 |
| Le, Y [48] | 2023 | Sage | 37078130 |
| Li, C [83] | 2022 | Front Public Health | 36225783 |
| Li, Z [34] | 2023 | Cancer Medicine | 36128836 |
| Liao, C [84] | 2023 | Diagnostics | 36832173 |
| Lin, Q [85] | 2021 | Nature | 33608560 |
| Liu, K [86] | 2023 | Cancers | 37296938 |
| Liu, S [87] | 2022 | Cancer Management and Research | 35018121 |
| Liu, WC [45] | 2021 | Cancer Management and Research | 34849027 |
| Liu, WC [88] | 2021 | Cancer Medicine | 33709570 |
| Liu, X [39] | 2021 | Frontiers | 34912716 |
| Liu, Y [89] | 2021 | BMC Medical Imaging | 37424672 |
| Long, Z [90] | 2023 | Frontiers | 36890826 |
| Massaad, E [91] | 2021 | Journal of Neurosurgery | 33932935 |
| Meares, C [92] | 2019 | Journal of Bone Oncology | 30847272 |
| Miao, S [93] | 2023 | Cancer Biomarkers | 38043007 |
| Motohashi, M [94] | 2023 | Spine | 38084012 |
| Noguchi, S [95] | 2022 | European Radiology | 35394186 |
| Ogura, K [96] | 2017 | Clinical Orthopaedics and Related Research | 28560532 |
| Overmann, AL [97] | 2020 | Journal of Orthopaedic Research | 32492213 |
| Ozkan, K [98] | 2023 | Indian Journal of Cancer | 36861722 |
| Park, JH [99] | 2024 | The Bone & Joint Journal | 38295850 |
| Piccioli, A [100] | 2015 | BMC Cancer | 25998535 |
| Shah AA [35] | 2021 | The Spine Journal | 33798728 |
| Skalitzky, MK [36] | 2022 | Journal of Surgical Oncology | 34608991 |
| Son, HJ [101] | 2023 | Heliyon | 38076046 |
| Su, CC [32] | 2023 | Journal of the American Academy of Orthopaedic Surgeons | 37192422 |
| Thio, QCBS [7] | 2020 | Clinical Orthopaedics and Related Research | 31651589 |
| Tokuda, O [102] | 2014 | Annals of Nuclear Medicine | 24573796 |
| Truong, VT [103] | 2023 | Asian Spine Journal | 37408290 |
| Tseng, TE [104] | 2022 | Clinical Orthopaedics and Related Research | 34491920 |
| Ujike, T [105] | 2022 | International Journal of Clinical Oncology | 35119579 |
| Wang, D [106] | 2023 | Journal of Bone Oncology | 37228896 |
| Wang, H [107] | 2023 | Medical Physics | 37966123 |
| Wang, H [108] | 2023 | Journal of Bone Oncology | 37670740 |
| Wang, J [109] | 2017 | Computers in Biology and Medicine | 28364643 |
| Wang, Y [95] | 2024 | Current Medical Imaging | 38258593 |
| Wang, Z [111] | 2021 | Pain and Therapy | 33740239 |
| Wang, Z [112] | 2016 | Oncotarget | 26871471 |
| Wuestemann, J [113] | 2020 | Cancers | 32957650 |
| Xiong, F [114] | 2022 | Frontiers in Cell and Developmental Biology | 36568969 |
| Xiong, Y [41] | 2023 | European Radiology | 37060446 |
| Yang, JJ [37] | 2021 | The Spine Journal | 33545371 |
| Yen, HK [115] | 2022 | Radiotherapy and Oncology | 36067909 |
| Zegarek, G [38] | 2022 | Acta Neurocirurgica | 35925406 |
| Zhong, G [116] | 2023 | The Spine Journal | 36706921 |
| Zhong, X [117] | 2023 | Scientific Reports | 37880320 |

**Supplementary table 2.** UPM scores of development studies on survival prediction (n=18)

| First author | Single, Bi-, or Multi-institutional | Study design | Sample size | Internal validation | Internal AUC | Calibration assessed | Weighted External AUC | WBC | UPM Score | TRIPOD Completeness |
| --- | --- | --- | --- | --- | --- | --- | --- | --- | --- | --- |
| Alcorn, SR [51] | Single | Retrospective | 397 | Yes | 0.82  3m: 0.83  6m: 0.81  12m: 0.81 | No | 0,79 [66] | No | 6 (Fair) | 76% |
| Anderson, AB [53] | Multi | Retrospective | 438 | Yes | 0,79  12m: 0.76  24m: 0.73  36m: 0.86  48m: 0.82  60m: 0.79  120m: 0.79 | Yes | † | Yes | 9 (Good) | 84% |
| Cui, Y [63] | Multi | Retrospective | 19.887 | Yes | 0.82 | No | † | No | 8 (Good) | 89% |
| Cui, Y [64] | Multi | Retrospective | 1.010 | Yes | 0.79 | No | 0.75 | Yes | 9 (Good) | 73% |
| Forsberg, JA [69] | Single | Retrospective | 189 | Yes | 0.91  3m: 0.89  12m: 0.93 | Yes | † | No | 7 (Good) | 81% |
| Forsberg, JA [46] | Single | Retrospective | 1.004 | Yes | 0.76  1m: 0.76  6m: 0.76 | Yes | 0.80  1m: 0.77  3m: 0.80  6m: 0.83  12m: 0.80 [96] | Yes | 10 (Good) | 75% |
| Forsberg, JA [67] | Single | Retrospective | 189 | Yes | 0.84  3m: 0.85  12m: 0.83 | No | 0.78  3m:0.79  12m: 0.76 [68,100] | No | 7 (Good) | 70% |
| Huang, Z [76] | Multi | Retrospective | 5.973 | Yes | 0,78 | No | † | No | 7 (Good) | 70% |
| Janssen, SJ [12] | Bi | Retrospective | 927 | Yes | 0.73  1m: 0.72  3m: 0.75  12m: 0.73 | No | † | No | 5 (Fair) | 93% |
| Karhade, AV [3] | Multi | Retrospective | 4.304 | Yes | 0.84 | Yes | 0.78 [32] | Yes | 13 (Excellent) | 85% |
| Karhade, AV [4] | Bi | Retrospective | 732 | Yes | 0.84  3m: 0.83  12m: 0.85 | Yes | 0.80  3m: 0.78  12m: 0.81  [31,33–37,37,38] | Yes | 12 (Excellent) | 73% |
| Karhade, AV [47] | Multi | Retrospective | 1.790 | Yes | 0.78 | Yes | † | Yes | 10 (Good) | 75% |
| Le, Y [48] | Multi | Retrospective | 1.532 | Yes | 0.74  12m: 0.71  36m: 0.76 | Yes | 0.74  12m: 0.81  36m: 0.67  [48] | No | 11 (Good) | 70% |
| Li, C [83] | Multi | Retrospective | 15.129 | Yes | 0.80  12m: 0.81  36m: 0.79  60m: 0.79 | No | † | No | 7 (Good) | 60% |
| Long, Z [90] | Multi | Retrospective | 1.995 | Yes | 0.78 | Yes | 0.76 [90] | No | 11 (Good) | 67% |
| Thio, QCBS [7] | Bi | Retrospective | 1.090 | Yes | 0.86  3m: 0.87  12m: 0.85 | Yes | 0.78  3mo: 0.78  6mo: 0.78  [2,99,104] | Yes | 12 (Excellent) | 84% |
| Xiong, F [114] | Multi | Retrospective | 16.189 | Yes | 0.82 | Yes | † | No | 10 (Good) | 63% |
| Zhong, X [117] | Multi | Retrospective | 283.373 | Yes | 0.94 | Yes | † | No | 10 (Good) | 64% |

**Supplementary table 3.** UPM scores of imaging studies (n=37)

| First author | Single, Bi-, or Multi-institutional | Study design | Sample size | Internal validation | Internal AUC | Calibration assessed | Weighted External AUC | WBC | UPM Score | CLAIM Completeness |
| --- | --- | --- | --- | --- | --- | --- | --- | --- | --- | --- |
| Ceranka, J [56] | Single | Retrospective | 27 | Yes | † | Yes | † | No | 4 (Fair) | 71% |
| Chang, CY [57] | Single | Retrospective | 600 | Yes | † | No | † | Yes | 5 (Fair) | 71% |
| Chen, K [59] | Single | Retrospective | 217 | Yes | 0.77 | No | † | No | 4 (Fair) | 71% |
| Chen, YY [60] | Single | Retrospective | 200 | Yes | † | No | † | No | 3 (Fair) | 57% |
| Chiu, JS [61] | Single | Retrospective | 111 | Yes | 0.88 | Yes | † | No | 6 (Fair) | 55% |
| Chmelik, J [62] | Single | Retrospective | 31 | Yes | 0.79 | No | † | No | 3 (Fair) | 64% |
| Duan, S [65] | Multi | Retrospective | 121 | Yes | 0.98 | No | † | Yes | 7 (Good) | 57% |
| Hallinan, JTPD [40] | Multi | Retrospective | 196 | Yes | † | No | † | No | 5 (Fair) | 79% |
| Hallinan, JTPD [49] | Multi | Retrospective | 257 | Yes | 0.91 | No | 0.94 | No | 10 (Good) | 74% |
| Hallinan, JTPD [71] | Multi | Retrospective | 185 | Yes | 0.97 | No | † | No | 7 (Good) | 76% |
| Hammon, M [72] | Single | Retrospective | 50 | Yes | † | No | † | No | 2 (Poor) | 40% |
| Hoshiai, S [73] | Multi | Retrospective | 80 | No | † | No | † | No | 2 (Poor) | 57% |
| Hsieh, T [75] | Multi | Retrospective | 19.041 | Yes | † | No | † | No | 6 (Fair) | 50% |
| Huo, T [77] | Multi | Retrospective | 126 | Yes | 0.87 | No | 0.88 | No | 9 (Good) | 69% |
| Ibrahim, A [78] | Multi | Retrospective | 1.367 | Yes | † | No | 0.89 | No | 9 (Good) | 48% |
| Kikushima, S [80] | Multi | Retrospective | 399 | Yes | 0.86 | No | † | No | 8 (Good) | 50% |
| Koike, Y [81] | Single | Retrospective | 79 | Yes | 0.94 | No | † | No | 4 (Fair) | 50% |
| Lang, N [82] | Multi | Retrospective | 61 | Yes | † | No | † | No | 4 (Fair) | 50% |
| Liao, C [84] | Multi | Retrospective | 920 | No | † | No | † | No | 4 (Fair) | 55% |
| Lin, Q [85] | Multi | Retrospective | 251 | Yes | 0.99 | No | † | No | 7 (Good) | 57% |
| Liu, K [86] | Multi | Retrospective | 295 | Yes | 0.77 | No | † | No | 6 (Fair) | 67% |
| Liu, S [87] | Multi | Retrospective | 621 | No | † | No | † | No | 4 (Fair) | 60% |
| Liu, X [39] | Multi | Retrospective | 955 | Yes | 0.82 | No | 0.91 | No | 11 (Good) | 83% |
| Liu, Y [89] | Single | Retrospective | 3.352 | Yes | 0.86 | No | † | No | 6 (Fair) | 45% |
| Miao, S [93] | Single | Retrospective | 431 | Yes | 0.92 | No | † | No | 6 (Fair) | 55% |
| Motohashi, M [94] | Single | Retrospective | 447 | Yes | 0.78 | No | † | Yes | 5 (Fair) | 67% |
| Noguchi, S [95] | Single | Retrospective | 832 | Yes | † | No | † | No | 4 (Fair) | 74% |
| Son, HJ [101] | Single | Retrospective | 4.626 | Yes | 0.82 | No | † | No | 6 (Fair) | 60% |
| Tokuda, O [102] | Single | Retrospective | 406 | Yes | † | No | † | No | 3 (Fair) | 31% |
| Ujike, T [105] | Single | Retrospective | 300 | Yes | 0.84 | No | † | No | 5 (Fair) | 36% |
| Wang, D [106] | Multi | Retrospective | 941 | Yes | † | No | † | No | 6 (Fair) | 40% |
| Wang, H [108] | Single | Retrospective | 81 | Yes | 0.98 | No | † | No | 4 (Fair) | 52% |
| Wang, H [107] | Single | Retrospective | 141 | Yes | 0.94 | No | † | No | 4 (Fair) | 48% |
| Wang, J [109] | Multi | Retrospective | 26 | Yes | † | No | † | No | 4 (Fair) | 55% |
| Wang, Y [110] | Single | Retrospective | 527 | Yes | 0.85 | No | † | No | 6 (Fair) | 57% |
| Wuestemann, J [113] | Multi | Retrospective | 4.702 | Yes | 0.89 | No | † | No | 8 (Good) | 38% |
| Xiong, Y [41] | Multi | Retrospective | 1.376 | Yes | 0.99 | No | 0.95 | No | 11 (Good) | 79% |

**Supplementary table 4.** UPM scores of ‘other’ models (n=11)

| First author | Single, Bi-, or Multi-institutional | Study design | Sample size | Internal validation | Internal AUC | Calibration assessed | Weighted External AUC | WBC | UPM Score | TRIPOD Completeness |
| --- | --- | --- | --- | --- | --- | --- | --- | --- | --- | --- |
| Albaradei, S [43] | Multi | Retrospective | 534 | Yes | 0.92 | No | † | Yes | 9 (Good) | 49% |
| Cai, WL [54] | Multi | Retrospective | 12.591 | Yes | 0.86 | No | † | No | 8 (Good) | 29% |
| Chavalparit, P [58] | Single | Retrospective | 167 | Yes | 0.90  3m: 0.85  6m: 0.94 | Yes | † | Yes | 6 (Fair) | 63% |
| Gao, L [44] | Single | Prospective | 1.043 | Yes | 0.87 | No | † | No | 9 (Good) | 64% |
| Gui, C [70] | Single | Retrospective | 95 | Yes | 0.88 | No | † | No | 4 (Fair) | 54% |
| Huang, CC [42] | Single | Retrospective | 2.768 | Yes | 0.82  1.5m: 0.84  3m: 0.84  12m: 0.77 | Yes | † | Yes | 9 (Good) | 89% |
| Liu, WC [45] | Multi | Retrospective | 207.137 | Yes | 0.96 | No | † | Yes | 9 (Good) | 53% |
| Liu, WC [88] | Multi | Retrospective | 17.138 | Yes | 0.92 | No | † | No | 8 (Good) | 49% |
| Wang, Z [111] | Single | Retrospective | 746 | Yes | 0.85 | No | † | No | 6 (Fair) | 48% |
| Wang, Z [112] | Single | Retrospective | 1.143 | Yes | 0.88 | No | † | No | 6 (Fair) | 50% |
| Yen, HK [115] | Single | Retrospective | 2.768 | Yes | 0.77  3m: 0.78  12m: 0.76 | Yes | † | Yes | 8 (Good) | 66% |
